# Survival is associated with complete response on MRI after neoadjuvant chemotherapy in ER-positive HER2-negative breast cancer

**DOI:** 10.1186/s13058-016-0742-0

**Published:** 2016-08-05

**Authors:** Claudette E. Loo, Lisanne S. Rigter, Kenneth E. Pengel, Jelle Wesseling, Sjoerd Rodenhuis, Marie-Jeanne T. F. D. Vrancken Peeters, Karolina Sikorska, Kenneth G. A. Gilhuijs

**Affiliations:** 1 Division of Diagnostic Oncology (Department of Radiology), The Netherlands Cancer Institute – Antoni van Leeuwenhoek Hospital, Plesmanlaan 121, Amsterdam, 1066 CX The Netherlands; 2 Division of Medical Oncology, The Netherlands Cancer Institute – Antoni van Leeuwenhoek Hospital, Plesmanlaan 121, Amsterdam, 1066 CX The Netherlands; 3 Division of Diagnostic Oncology (Department of Pathology) and Division of Molecular Pathology, The Netherlands Cancer Institute – Antoni van Leeuwenhoek Hospital, Plesmanlaan 121, Amsterdam, 1066 CX The Netherlands; 4 Division of Surgical oncology, The Netherlands Cancer Institute – Antoni van Leeuwenhoek Hospital, Plesmanlaan 121, Amsterdam, 1066 CX The Netherlands; 5 Department of Biostatistics, The Netherlands Cancer Institute – Antoni van Leeuwenhoek Hospital, Plesmanlaan 121, Amsterdam, 1066 CX The Netherlands; 6 Department of Radiology and the Image Science Institute, University Medical Center Utrecht, Utrecht, The Netherlands

**Keywords:** Breast cancer, Neoadjuvant chemotherapy, Magnetic resonance imaging, Recurrence-free survival, Estrogen receptor

## Abstract

**Background:**

Pathological complete remission (pCR) of estrogen receptor (ER)-positive/human epidermal growth factor receptor 2 (HER2)-negative breast cancer is rarely achieved after neoadjuvant chemotherapy (NAC). In addition, the prognostic value of pCR for this breast cancer subtype is limited. We explored whether response evaluation by magnetic resonance imaging (MRI) is associated with recurrence-free survival after NAC in ER-positive/HER2-negative breast cancer.

**Methods:**

MRI examinations were performed in 272 women with ER-positive/HER2-negative breast cancer before, during and after NAC. MRI interpretation included lesion morphology at baseline, changes in morphology and size, and contrast uptake kinetics. These MRI features, clinical characteristics and final pathology were correlated with recurrence-free survival.

**Results:**

The median follow up time was 41 months. There were 35 women with events, including 19 breast-cancer-related deaths. On multivariable analysis, age younger than 50 years (hazard ratio (HR) = 2.55, 95 % confidence interval (CI) 1.3, 5.02, *p* = 0.007), radiological complete response after NAC (HR = 14.11, CI 1.81, 1818; *p* = 0.006) and smaller diameters of washout/plateau enhancement at MRI after NAC (HR = 1.02, CI 1.00, 1.04, *p* = 0.036) were independently associated with best recurrence-free survival. Pathological response was not significant; HR = 2.12, CI 0.86, 4.64, *p* = 0.096.

**Conclusions:**

MRI after NAC in ER-positive/HER2-negative tumors may be predictive of recurrence-free survival. A radiological complete response at MRI after NAC is associated with an excellent prognosis.

## Background

Neoadjuvant chemotherapy (NAC) for breast cancer has been shown to be equally effective as postoperative chemotherapy in terms of disease-free and overall survival [1–4]. Several markers are routinely employed to predict treatment outcome and to select therapy [5–7]. The most frequently used include the estrogen receptor (ER), the progesterone receptor (PR) and the human epidermal growth factor receptor 2 (HER2). Three major breast cancer subtypes are easily distinguished by immunohistochemical assessment (IHC): triple-negative (ER, PR and HER2-negative), HER2-positive (HER2-positive (ER and PR may be positive or negative)) and ER-positive/HER2-negative (ER-positive, HER2-negative (PR may be positive or negative)) [8, 9]. These immunohistochemical subtypes correspond roughly to the molecular subtypes, basal-like, HER2-enriched and luminal, respectively [10]. Subtyping of typically heterogeneous breast cancer in these three groups may improve understanding of tumor response and outcome and may result in optimized strategies for patient-tailored treatment [11, 12].

Even within these subgroups, the response to and outcome after chemotherapy vary widely. Pathologically confirmed complete remission (pCR) or minimal disease [13, 14] after chemotherapy is associated with disease-free and overall survival [1, 2, 15, 16]. More recently, however, it has been shown that this relationship is absent for luminal A tumors [17], which comprise approximately half of the tumors that express the ER but which do not contain a HER2 gene amplification. Nevertheless, pCR is often used as a surrogate marker to predict long-term outcome in this subgroup. Of patients with ER-positive/HER2-negative tumors only a small fraction will achieve pCR, while the prognosis is better than that of triple-negative breast cancer [17]. Therefore pCR after NAC in ER-positive/HER2-negative tumors is certainly not a practical prognostic indicator. It is possible that dynamic contrast-enhanced magnetic resonance imaging (MRI), which visualizes functional properties of the tumor such as those associated with angiogenesis, may be used as a practical prognostic indicator.

The benefit of MRI over other imaging modalities for monitoring response during and after NAC has been extensively reported [18–21]. Also prediction of pathological response after NAC by MRI has been extensively studied [22–24]. A recent published study evaluated volumetric MRI for predicting recurrence-free survival after NAC in patients with breast cancer [25]. However, the role of MRI after NAC in predicting survival in patients with ER-positive/HER-2negative tumors in particular has not yet been completely assessed. The purpose of this study was to explore whether MRI is associated with recurrence-free survival after neoadjuvant chemotherapy in ER-positive/HER2-negative breast cancer.

## Methods

### Selection of patients

Patients between 18 and 70 years of age with pathologically proven invasive ER-positive/HER2-negative breast cancer >3 cm in size and/or at least one tumor-positive lymph node were offered NAC. All patients received NAC between January 2000 and June 2012, and all either took part in a single-institution clinical trial (approved by the Medical Research Ethics Committee of the Netherlands Cancer Institute), or were treated off study according to the standard arm of the trial [26, 27]. The institutional review board had approved the study protocols and informed consent was obtained from all patients. Only patients with ER-positive/HER2-negative tumors based on immunohistochemical assessment without a prior history of breast cancer were included in this analysis. Only patients who had undergone MRI before (baseline), during (after three courses) and after NAC and who underwent surgery after NAC were included.

### Treatment

Four different regimens of NAC were employed [26, 27]. Between 2000 and 2004 patients were randomized to receive either six cycles of treatment AC or six cycles of treatment AD, with AC being considered as standard treatment. AC consisted of doxorubicin 60 mg/m^2^ and cyclophosphamide 600 mg/m^2^ every three weeks, whereas patients in the AD arm were treated with six cycles of doxorubicin 50 mg/m^2^ and docetaxel 75 mg/m^2^. After 2004, patients started with three courses of ddAC (doxorubicin 60 mg/ m^−2^ and cyclophosphamide 600 mg/ m^−2^ on day 1, every 14 days, with PEG-filgrastim on day 2). When an unfavorable response was noted on MRI (defined as a reduction <25 % in the largest diameter of the tumor plateau/washout enhancement [28]) after three courses of treatment, chemotherapy was switched to a (theoretically) non-cross-resistant regimen. In such a case, three courses of ddAC were followed by three courses of docetaxel and capecitabine (DC, docetaxel 75 mg/ m^−2^ on day 1, every 21 days and capecitabine 2 × 1000 mg/ m^−2^ on days 1–14). In the case of a favourable response on MRI, chemotherapy was continued with three further courses of ddAC.

After the last course of chemotherapy, all patients underwent surgery (breast-conserving surgery or mastectomy with or without axillary lymph node dissection), post-operative external beam radiation therapy, and adjuvant endocrine therapy, according to standard guidelines.

### MRI and evaluation

Initially MRI was performed on a 1.5 T Magnetom Vision scanner with a dedicated bilateral phased array breast coil (Siemens, Erlangen, Germany). From April 2007 MRI was performed on a 3.0 T Achieva scanner with a dedicated 7-element sense breast coil (Philips Medical Systems, Best, The Netherlands). Images were acquired with the patient in the prone position and with both breasts imaged simultaneously. The standard dynamic protocol started with an unenhanced coronal 3D fast field echo (FFE) (thrive) sense T1-weighted sequence. A bolus (14 mL) of contrast containing gadolinium (0.1 mmol/kg) was administered intravenously at 3 mL/s using a power injector followed by a bolus of 30 mL of saline solution. Subsequently, dynamic imaging was performed in five consecutive series at 90-s intervals. The voxel size was 1.21 × 1.21 × 1.69 mm^3^ (1.5 T) or 1.1 × 1.1 × 1.2 mm^3^ (3.0 T). The following scanning parameters were used: acquisition time 90 s (1.5 T and 3.0 T); repetition time (TR)/echo time (TE): 8.1/4.0 (1.5 T) or 4.4/2.3 (3.0 T); flip angle 20° (1.5 T) or 10° (3.0 T); field of view (FOV) 310 (1.5 T) or 360 (3.0 T).

Breast MR images were interpreted using a viewing station that permitted simultaneous viewing of two series reformatted and linked in three orthogonal directions [29]. The viewing station displays all imaging series (unenhanced and contrast-enhanced), subtraction images at 90-s intervals and maximum intensity projection (MIP) of both breasts. The displayed images were also color coded, representing different levels and curve types of enhancement. Specifically, the color indicated the shape of the time-signal intensity (contrast enhancement) curve at each pixel location [30]: type I (i.e., persistent enhancement >10 % after the first post-contrast image), type II (i.e., plateau enhancement between −10 % and +10 % during late enhancement), and type III (i.e., washout kinetics resulting in >10 % signal decrease during late enhancement) [30]. These colors were coded yellow, light red and dark red, respectively, where initial enhancement (90 s) equaled or exceeded 100 % and green, light blue and dark blue, respectively, where initial enhancement was between 50 % and 100 %. The viewing station was developed in close collaboration with the breast radiologists at the Netherlands Cancer Institute. The radiologists have been using the system since 2000.

The MR images were assessed by four breast radiologists, who were unaware of the outcome. The patients were randomly distributed among the radiologists for assessment. The MR images before (baseline), during and after chemotherapy were analyzed by the same radiologist in one session to ensure interpretive consistency. Temporal and morphologic characteristics of contrast uptake were scored as previously described [28]. In short, tumor extent, morphology and relative enhancement were assessed during initial enhancement (90 s) and late enhancement (450 s) on all subsequent MRI scans.

The extent of the tumor was assessed by its largest diameter in three reformatted planes (sagittal, axial and coronal) at initial and at late (washout/plateau) enhancement separately. If a non-mass (diffuse) enhancement or multifocal disease was visible, the total area including non-enhancing breast tissue between lesions was measured on MIP images. The largest value of the three diameters was recorded. The percentage difference in largest tumor diameter between subsequent MRI scans was also assessed, both at initial and at late enhancement. Supported by the color coding, the area within the tumor with the strongest contrast uptake at initial and at late enhancement was determined. Measurement of the signal intensity (initial and late enhancement) was performed manually by placing a region of interest in the most malignant area (dark red) and moving the cursor in this area to find the most malignant values, in real time (in percentages). Morphology of the enhancing tumor was scored in three groups: unifocal mass, multifocal mass and non-mass (diffuse) enhancement [31]. On MRI during and after NAC the pattern of tumor reduction was denoted in five categories: shrinking mass, diffuse decrease, reduction to small foci, no enhancement and no change.

Complete absence of contrast enhancement in the original tumor bed on MRI after NAC was defined as radiological complete response. Consequently, evidence of small enhancing foci in the original tumor bed was considered as residual enhancing tumor.

### Histopathologic analysis

Prior to NAC at least three 14-G ultrasound-guided core biopsies of the breast tumor were taken. Subsequently, most tumors were marked with a radiopaque marker. ER and PR status were determined by immunohistochemical assessment and considered positive if ≥10 % of nuclei stained positive, and HER2 status was assessed by scoring the intensity of membrane staining. Tumors with a score of 3+ (strong homogeneous staining) were considered positive. In the case of 2+ scores (moderate homogeneous staining) chromogenic in situ hybridization (CISH) was used to determine HER2 amplification (gene copy number of six or more per tumor cell). For this study ER-positive/HER2-negative tumors were selected.

### Pathologic response

Three common definitions of pCR were used: (1) no residual invasive tumor in the breast (ypT0/is) [15, 32], (2) no residual invasive tumor in the breast or axilla (ypT0/isN0) [33] and (3) a near-complete response, indicating the presence of only a small number of scattered tumor cells in the breast (ypT < mic) [14].

### Statistical analysis

The primary endpoint was recurrence-free survival (RFS), defined according to the standardization of events and endpoints (STEEP) criteria [34]. According to this definition an event is either a local, regional or distant breast cancer recurrence or death due to any cause. Second primaries (including contralateral breast cancer) were not considered an event. The final data were collected in September 2014, and patients for whom no event had occurred were censored at the last date of being seen alive.

The median length of follow up was calculated using the reverse Kaplan-Meier approach. Patient characteristics are presented in tables as medians (percentiles) for continuous variables and frequencies for categorical variables. All clinical variables were analyzed as categorical predictors (Table 1). The MRI characteristics were analyzed as categorical variables (Table 2) or continuous variables (Table 3). For the categorical predictors, the first mentioned category was taken as a reference and the hazard ratio (HR) compares the subsequent categories to the reference. For the continuous predictors, the HR represents a change in hazard for one unit change in the predictor.

**Table 1:** Univariable Cox proportional hazard analysis of relationship between clinical variables and recurrence-free survival

				Recurrence-free survival		
Variable		Number of patients	Number of events	*P* value	Hazard ratio	95 % CI
Tumor (T) stage prior to NAC				0.731		
T1		28	1			
T2		149	19		2.42	0.32, 18.16
T3		79	12		2.7	0.35, 20.99
T4		16	3		2.91	0.30, 28.09
Node (N) stage prior to NAC				0.558		
Negative		55	6			
Positive		217	29		1.29	0.54, 3.11
Clinical stage				0.847		
II		185	24			
III		86	11		0.93	0.46, 1.91
Unknown		1				
Age				**0.008**		
≤50 years at diagnosis		177	17			
>50 years at diagnosis		95	18		2.49	1.28, 4.85
Menopausal status				**0.017**		
Premenopausal		161	15			
Perimenopausal		16	2		1.42	0.32, 6.24
Postmenopausal		91	18		2.74	1.38, 5.46
Unknown		4				
Histology^a^				0.835		
Adenocarcinoma, *n*		18	3			
Ductal carcinoma		207	27		1.39	0.42, 4.62
Lobular carcinoma		39	4		0.93	0.21, 4.16
Other		8	1		1.08	0.11, 10.42
Progesterone receptor^a^				0.199		
Negative		76	13			
Positive		192	21		0.63	0.31, 1.26
Unknown		4				
Tumor grade^a^				0.14		
Good		28	2			
Moderate		117	16		3.57	0.8, 15.93
Poor		32	5		3.52	0.66, 18.73
Unknown		95				
Chemotherapy regimen				0.89		
ddAC		167	20			
AC-CD		77	8		1.23	0.54, 2.83
AD		14	4		0.79	0.25, 2.55
CD		13	3		1.33	0.39, 4.51
Unknown		1				
Pathologic response						
ypT0/isypN0:	No	261	35	0.41		
	yes	11	0		0.37	0, −^b^
ypT0/is:	No	251	34	0.29		
	yes	21	1		0.39	0.05, 2.88
ypT < mic:	No	221	28	0.91		
	yes	51	7		0.95	0.42, 0.91

Univariable Cox model for clinical and pathologic parameters of recurrence-free survival. ^a^Determined on pre-chemotherapy ultrasound-guided biopsy. ^b^− CI boundary could not be estimated. *NAC* neoadjuvant chemotherapy, *CI* confidence interval , *(dd)AC* (dose-dense) cyclophosphamide and doxorubcin, *CD* capecitabine and docetaxel, *AD* doxorubcin and docetaxel, *ypT0/isypN0* no residual invasive tumor in breast and axilla, *ypT0/is* no residual invasive tumor in the breast, *ypT < mic* few scattered tumor cells in the breast. Numbers in bold are significant values

**Table 2:** Univariable Cox proportional hazard analysis of relationship between MRI variables and recurrence-free survival

	Number of	Number of	Recurrence free survival		
Variable	patients	events	*P* value	Hazard ratio	95 % CI
Lesion morphology baseline MRI			0.612		
Mass unifocal	91	10			
Mass multifocal	96	12		1.29	0.71, 3.70
Non mass (diffuse)	77	13		1.60	0.57, 3.01
Mass and non mass	8	0		4.38	0.03, 41.66
Pattern of reduction at MRI after NAC			**0.029**		
No change	23	4			
Shrinking mass	96	10		0.56	0.19, 1.89
Diffuse decrease	56	10		0.78	0.27, 2.64
Small foci	53	11		0.95	0.34, 3.19
No enhancement	44	0		0.06	0, 0.53
Dynamic curve type after NAC			**0.008**		
No enhancement	44	0			
Continuous	89	16		13.54	1.83, 1728.03
Plateau	82	5		7.46	0.84, 980.87
Washout	57	14		17.59	2.35, 2248.46
Radiological complete response			**0.004**		
Yes	44	0			
No	228	35		12.81	–^a^, 1621.10
RECIST evaluation MRI initial after NAC - baseline			**0.009**		
No enhancement after NAC	44	0			
Part Rem (LD initial↓ ≥30 %)	154	23		11.63	–^a^, 1477.68
NR (LD initial↓ <30 %)	74	12		16.53	2.17, 2119.55
RECIST evaluation MRI initial after NAC – during			**0.037**		
No enhancement during and after NAC	10	0			
No enhancement after NAC	36	0		0.44	0, 80.78
Part Rem (LD initial↓ ≥30 %)	82	11		3.99	0.52, 513.45
NR (LD initial ↓ <30 %)	144	24		4.95	0.68, 629.57
RECIST evaluation MRI late after NAC - baseline			0.05		
No washout/plateau baseline and after NAC	8	0			
No washout/plateau after NAC	136	17		3.08	0.41, 394.53
Part Rem (LD late ↓ ≥30 %)	93	12		3.54	0.46, 455.8
NR (LD late ↓ <30 %)	35	6		11.57	1.31, 1525.99
RECIST evaluation MRI late after NAC - during			0.46		
No plateau/washout during and after NAC	61	6			
No plateau/washout after NAC	84	11		1.17	0.43, 3.17
Part Rem (LD late ↓ ≥30 %)	58	9		1.65	0.59, 4.64
NR (LD late ↓ <30 %)	69	9		2.08	0.74, 5.87

Univariable Cox proportional hazard analysis of magnetic resonance imaging (MRI) variables with recurrence-free survival. ^a^CI boundary could not be estimated. *CI* confidence interval, *LD* largest diameter, *Part Rem* partial remission, *initial* enhancement 90 s, *late* washout/plateau enhancement 450 s, *MRI* magnetic resonance imaging, *NAC* neoadjuvant chemotherapy, *NR* non responder, *RECIST* response evaluation criteria in solid tumors. Arrow (↓) indicates decrease. Numbers in bold are significant values

**Table 3:** Univariable Cox proportional hazard analysis of relationship between continuous MRI variables and recurrence-free survival

		Recurrence-free survival		
MRI variable	Median	*P* value	Hazard ratio	95 % CI
Baseline (before NAC)				
Largest diameter MIP/initial enhancement (90 s)	43 mm	0.238	1,009	0.994, 1.024
Largest diameter plateau/washout enhancement (450 s)	33 mm	**0.027**	1,017	1.002, 1.033
Initial enhancement (90 s) %	152 %	0.326	0.997	0.99, 1.003
Late enhancement (450 s) %	−13 %	0.947	0.999	0.967, 1.032
During NAC				
Largest diameter MIP/initial enhancement (90s)	30 mm	0.155	1,011	0.996, 1.026
Largest diameter plateau/washout enhancement (450 s)	17 mm	**0.006**	1,024	1.007, 1.041
Initial enhancement %	135 %	0.993	1.00	0.995, 1.005
Late enhancement %	−4 %	0.600	0.995	0.975, 1.015
After NAC				
Largest diameter MIP/initial enhancement (90s)^a^	22 mm	0.140	1.01	1.00, 1.03
Largest diameter plateau/washout enhancement (450 s)	0 mm	**0.003**	1.03	1.01, 1.051
Initial enhancement %	100 %	0.057	1,005	1.00, 1.011
Late enhancement %	9 %	0.815	0.998	0.983, 1.014
Percent change after NAC - baseline NAC %				
Largest diameter MIP/initial enhancement (90 s)^b^	−40 %	0.280	1.01	0.99, 1.02
Largest diameter plateau/washout enhancement (450 s)^c^	−100 %	**0.021**	1,013	1.002, 1.024
Percent change after NAC - during NAC %				
Largest diameter MIP/initial enhancement (90 s) mm^b^	−30 %	0.290	1.01	0.99, 1.02
Largest diameter plateau/washout enhancement (450 s)^c^	−61 %	0.066	1,008	0.999, 1.017

^a^Patients with largest diameter 0 mm were excluded; ^b^patients with change −100 % were excluded; ^c^patients without washout/plateau on both scans were excluded. *MRI* magnetic resonance imaging, *CI* confidence interval, *NAC* neoadjuvant chemotherapy, *MIP* maximum intensity projection. Numbers in bold are significant

The clinical and MRI characteristics were first tested for association with the outcome in univariable Cox models. Next, the significant and clinically relevant parameters were analyzed jointly in a multivariable Cox model. When at least one of the analyzed subgroups had no events, the Cox regression with Firth’s penalized likelihood was used for the estimation of the hazard ratios. Confidence intervals were then computed using profile likelihood. This technique has been implemented in the R package coxphf.

The optimal cut points and their significance for the continuous variables were estimated using maximally selected rank statistics as implemented in the R package maxstat. Variables for which the *p* value was <0.05 were considered significant. The final model was built by combining statistical evidence (significant *p* values) and clinical relevance (age, pathological response). All statistical analyses were performed using R software (version 3.1.0) or SPSS (version 20).

## Results

Between January 2000 and June 2012 428 patients with ER-positive/HER2-negative breast cancer were registered in the NAC breast database of our institute. Of these, 279 patients had response evaluation with MRI (before, during and after), underwent surgery and had no distant metastasis. Seven patients were excluded; four because of a history of breast cancer, two because of technically inadequate MRI, and one patient because she was found to have HER2-positive breast cancer. The majority of the 272 women were premenopausal, had invasive ductal carcinoma, positive nodal stage prior to NAC and tumor stage T2 tumors (Table 1). The median (range) of the measurements of the largest diameter of the initial tumor on MRI was 4.3 cm (1.0–11.5). The median age at diagnosis was 47 years (range 19–68). The median follow-up time was 41 months (3.4 years).

**Fig. 1:**
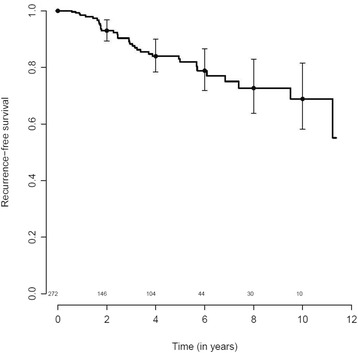
Recurrence-free survival among 272 patients with estrogen receptor (ER)-positive/human epidermal growth factor receptor 2 (HER2)-negative breast cancer after neoadjuvant chemotherapy (*solid line*), and the 95 % confidence interval. Numbers of patients at risk are shown above the *x-axis*

There were 35 women with an event; 31 women had distant metastases, 2 had additional local/regional recurrence, one only a local/regional recurrence and one patient died without any recurrence reported. There were 20 deaths: 19 breast-cancer-related deaths and 1 death due to another malignancy. The RFS for the study group is shown in Fig. 1.

### Univariable Cox model for clinical and pathological parameters

**Fig. 2:**
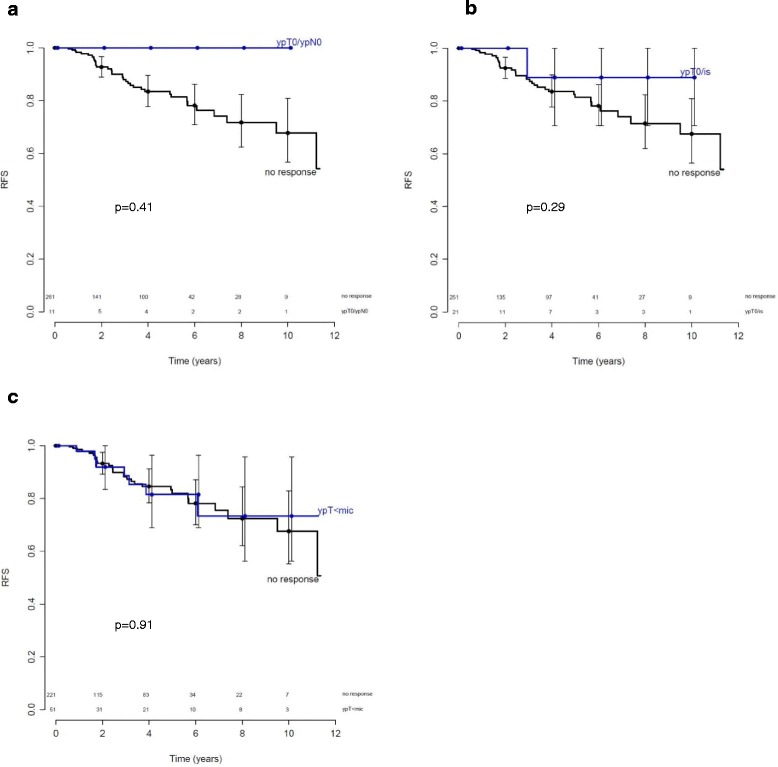
Kaplan-Meier curves for recurrence-free survival (*RFS*) in relation to pathologic response after neoadjuvant chemotherapy in patients with estrogen-receptor-positive tumors. The *solid line* indicates patients with no response. Numbers of patients at risk for each group are shown above the *x-axis*. **a** *Blue line* indicates no residual invasive tumor in the breast and axilla (*ypT0N0*) (*p* = 0.41); **b** *blue line* indicates no residual invasive tumor in the breast (*ypT0/is*) (*p* = 0.29); **c** *blue line* indicates only a small number of scattered tumor cells in the breast (*ypT < mic*, i.e., a near-complete response) (*p* = 0.91)

Among the clinical and pathological parameters, postmenopausal status (HR = 2.73, *p* = 0.04) and age over 50 years (HR = 2.49, *p* = 0.01) were associated with worse RFS (Table 1). pCR, according to any investigated definition, was not associated with improved RFS (Table 1, Fig. 2). Twenty-one patients (7.7 %) achieved an ypT0/is of the primary tumor after NAC. Only one recurrence was found in this group (p = 0.29). Eleven (4 %) patients had no residual invasive tumor in the breast or axilla (ypT0/isypN0) after NAC. In this group, no events occurred (*p* = 0.41). Also, a near pCR (a few scattered tumor cells in the breast (ypT < mic)) was observed in 51 patients (seven events), which was not associated with RFS (*p* = 0.91). Kaplan-Meier curves for the pathologic response are shown in Fig. 2.

### Univariable Cox model for MRI parameters

**Fig. 3:**
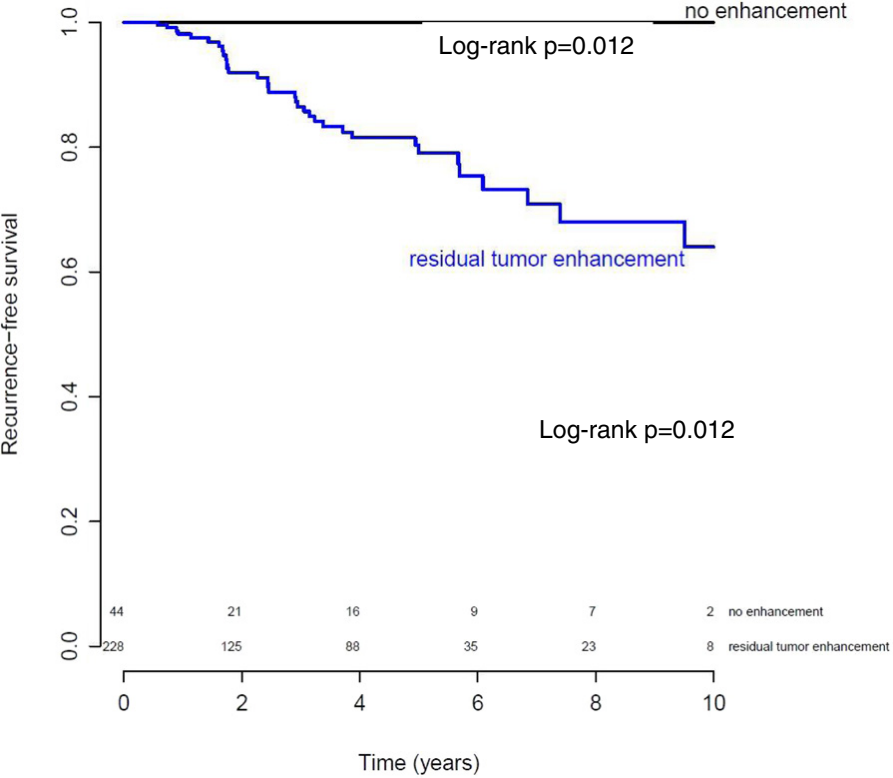
Kaplan-Meier curves for recurrence-free survival (RFS) of patients with estrogen-receptor-positive tumors based on radiological complete response (*black line* no enhancement) and those with residual enhancement (*blue line*) identified on magnetic resonance imaging after neoadjuvant chemotherapy. Log-rank test *p* = 0.012. Numbers of patients at risk in each group are shown above the *x-axis*

No tumor enhancement (i.e., a radiological complete response) after NAC (HR = 12.81, *p* = 0.004) was significantly associated with superior RFS (Table 2). Forty-four of the 272 patients (16.2 %) achieved a radiological complete response after NAC as identified on MRI. No events were found in this group. Kaplan-Meier curves for patients with ER-positive/HER2-negative breast cancer show significant difference in RFS between patients with a radiological complete response and those with residual enhancement on MRI (log-rank *p* = 0.012; Fig. 3).

**Fig. 4:**
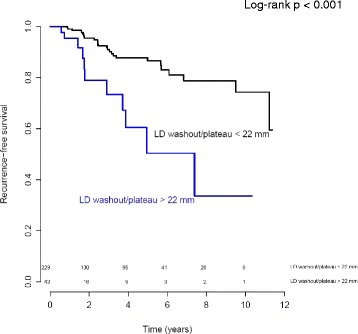
Kaplan Meier curve for recurrence-free survival of patients with ER-positive tumors with washout/plateau enhancement smaller than 22 mm (*black line*) and those with a diameter of washout/plateau larger than 22 mm (*blue line*) after neoadjuvant chemotherapy on magnetic resonance imaging. *LD* largest diameter. Numbers of patients at risk in each group are shown above the *x-axis*

Also the largest diameter of the region with washout/plateau (late) enhancement was associated with RFS on baseline MRI (HR = 1.017, *p* = 0.027), during NAC (HR = 1.024, *p* = 0.006) and after NAC (HR = 1.03, *p* = 0.003) (Table 3). The most significant cut off for the largest diameter of washout/plateau enhancement after NAC was estimated for the value 22 mm. Log-rank test *p* value <0.001 (Fig. 4). In addition, the percent change in the largest diameter of the region with washout/plateau enhancement between baseline and after NAC (HR = 1.013, *p* = 0.021) was associated with RFS (Table 3).

### Multivariable analysis

In the multivariable analysis we fitted a Cox model including radiological complete response after NAC, the largest diameter of washout/plateau on MRI after NAC, the patient’s age and pathological response (ypT < mic).The first three predictors remained statistically significant with HR of 14.11 (1.8–1818, *p* = 0.006), 1.02 (1.00–1.04, *p* = 0.036) and 2.55 (1.3–5.02, *p* = 0.007), respectively. Pathological response did not remain significant; HR = 2.12 (0.86–4.64, *p* = 0.096).

## Discussion

In a series of 272 consecutive patients with luminal (ER-positive/HER2-negative) breast cancer, radiological complete remission assessed on MRI after NAC was associated with significantly improved RFS after NAC. All of the 44 patients (16 %) with radiological complete response remained free of disease during follow up.

This finding may be of clinical importance. Luminal breast cancer is the most common breast cancer and represents approximately 2/3 of all cases. Patients with luminal tumors only rarely achieve pCR. In this study, only 8 % (21/272) achieved pCR in the breast and even fewer patients (4 % (11/272)), achieved pCR in the breast and axilla. In our study neither pCR (i.e., ypT0is or ypT0isN0) nor near-pCR was predictive of improved RFS. These findings are in accordance with previously published work [17]. Also other studies showed that pCR is not a suitable surrogate endpoint for patients with ER-positive/HER2-negative grade 1 or 2 (luminal A) breast cancer [35, 36].

We investigated the potential of MRI to predict recurrence-free survival. MRI after completion of chemotherapy was found to be of particular prognostic value in the current study. Apparently, the lack of enhancement on MRI, which provides information about functional properties of the tumor, is associated with prognosis in slowly proliferating tumors, but pCR is not.

Many studies have evaluated the role of MRI after NAC as a diagnostic tool to serve as a surrogate for final pathology [37–39]. The majority of studies have focused on the correlation between tumor size as assessed by MRI and that identified on pathology assessment to validate MRI as a tool to detect residual disease and to guide surgical planning. In terms of tumor size, MRI may underestimate or overestimate compared to pathology assessment, resulting in false-negative and false-positive results [40, 41]. Other studies have shown that a complete response after neoadjuvant chemotherapy identified on MRI is associated with the presence of residual tumor pathology assessment in 26–56 % of cases [26, 42]. More recent studies have indicated that the accuracy of MRI in estimating tumor size after neoadjuvant chemotherapy varies with breast cancer subtype and tumor morphology [22, 24, 39, 43]. The best accuracy is achieved in HER2-positive and triple-negative tumors [22, 24, 39]. We found that a radiological complete response in ER-positive breast cancer is associated with an excellent prognosis. However in 36 % (16/44) of these cases, there was (microscopic) residual tumor on the final pathology assessment.

We used a very strict definition of a radiological complete response in which even small enhancing foci in the original tumor bed are considered as residual tumor. Especially in diffuse tumors (non-mass enhancement) that disintegrate into (very) small foci the radiological assessment can be challenging and in clinical practice small enhancing foci may occasionally be interpreted incorrectly as a radiological complete response. We have observed such interpretation discrepancies between the retrospective dedicated review of our study and the clinical routine MRI assessment. For future validation studies it will be important to maintain the strict definition of radiological complete response.

The policy of changing the chemotherapy regimen in the case of an unfavorable MRI response during NAC could have led to an increase in the (radiological) complete remission rate in our study. This was certainly the objective of the policy, but whether this really succeeded needs to be further studied in controlled trials. We assumed that a larger reduction in tumor size on MRI could correlate with a smaller volume of residual tumor, but it could also serve as a measure of chemotherapy sensitivity. The latter could be critically important for the likelihood that micro-metastatic disease has been eradicated or reduced, which is the primary objective of NAC. The differences between radiological complete remission (CR) and pCR in this respect, include the more frequent occurrence of radiological CR in this type of tumor and perhaps the higher likelihood of radiological CR in tumor subtypes that tend to recur less often or later than others. Although a detailed subgroup analysis could not be performed due to the limited number of patients, there was no indication that the association between radiological CR and RFS was different for different chemotherapy regimens or between patients who did and those who did not cross over to a different chemotherapy regimen (Table 1).

The value of MRI with or without prognostic markers such as those derived from pathology assessment is yet unclear when it comes to predicting disease-free survival of patients with ER-positive/HER2-negative breast cancer. A few studies have investigated the predictive role of MRI in breast cancer survival after NAC without using a distinction in subgroups [44–46]. In a relatively small study group of 58 patients with a short median follow up of 33 months, Partridge et al. showed that initial MRI volume before NAC, and final change in MRI volume were significant predictors of RFS [44]. Yi et al. evaluated 158 breast cancer patients with MRI before and after NAC. They concluded that a smaller reduction in tumor volume and a smaller reduction in washout component, assessed with computer-aided evaluation, were associated with worse RFS [45].

Jafri et al. evaluated the optimal threshold for measuring functional tumor volume in 64 patients. They concluded that functional tumor volume is able to predict RFS and could be used as a biomarker [46]. These three studies did not report how many patients achieved radiological or clinical complete response on MRI, nor did they analyze breast cancer subgroups.

A more recent published study (ACRIN 6657) noted that functional tumor volume (tumor volume percent enhancement >70 %) after NAC is a strong predictor of RFS in breast cancer [25]. Their Kaplan-Meier analyses performed by subtype suggest that the ability of functional tumor volume to discriminate differences differs per breast cancer subtype. After NAC, a greater RFS separation was found in 78 ER-positive/HER2-negative and 41 HER2-positive breast cancers than in the whole group. Instead of volumetric measurements, we assessed the largest diameter at initial (MIP, 90 s) and late (washout/plateau) enhancement on MRI. In accordance with Yi et al. we found that the largest diameter of washout/plateau and the change in this diameter are significantly associated with RFS in our subset of ER-positive breast cancer. However, in daily clinical practice a radiological complete response is a more straightforward and potentially a more reproducible measure to identify patients with ER-positive/HER2-negative breast cancer who have a good prognosis. On the other hand, in patients with residual enhancement on MRI, and who thus may have a less favorable prognosis, the largest diameter of washout/plateau enhancement may be used to decide if additional chemotherapy is required. For this study population the most significant cut off was estimated for a largest diameter of 22 mm. However, before we can actually use this value we need to validate this in a larger study group, preferably with longer follow up.

Our study has some limitations. These involve potential suboptimal selection of groups and differences in chemotherapy regimens. The study ran for an extensive period of time (2000–2012). During this time, the 1.5 T MRI scanner was replaced by a 3 T scanner, and the MRI scan protocol was amended to standard clinical care. Care was taken to align the MRI protocols over time between 1.5 T and 3 T as much as possible, but minor differences could not be avoided in voxel size and FOV. During the study period the temporal resolution and methods used to analyze the images remained unchanged. Although we have no indication that this is the case, one can never be certain that small differences in scan protocols may affect the results in some way. This is a limitation that is difficult to avoid in longer-running radiological studies such as those presented here, given the rapid developments in MRI technology that inevitably find their way into daily clinical practice. Nonetheless, despite these differences, we were still able to demonstrate significant associations. In addition, the MRI measurements were performed interactively on the basis of automatically calculated color overlay images by different radiologists. Even though the measurements (largest diameter, ROI placement for relative enhancement percentage) were carried out carefully by dedicated breast radiologists and according to protocol this manual procedure is to a certain extent subjective and can lead to potential bias. Although more recent methods of volumetric assessment may further reduce subjectivity, it is difficult to avoid it altogether due to empirical adjustments of parameters such as percent-enhancement thresholds and placement of the region of interest [25].

Although the total study group is relatively large, only 35 recurrences occurred during a follow-up time that is relatively brief for ER-positive/HER2-negative (luminal) tumors. This resulted in wide confidence intervals for the hazard ratios. In this study the tumor grade determined on the biopsy was known in only 177 (65 %) patients. As a result we were not able to allow additional stratification in luminal A and luminal B tumors. Ideally, subtyping would also have been based on gene expression rather than on immunohistochemical assessment, and the median follow up would have been longer, with more recurrences available for analysis. On the other hand, the predictive effect of a radiologic complete response may be especially clear in the first 5 years after NAC. The Oxford overview has shown that chemotherapy prevents recurrences within the first 5 years, while the preventive effect of endocrine treatment, which is at least as important in luminal tumors, extends beyond 10 years [47]. As a result, effective chemotherapy may prevent early relapse, seen after limited follow up, when the endocrine treatment effect is not yet dominant.

Another limitation is that different dedicated breast radiologists in a single institution using strict criteria assessed the results. As a result, we were not able to evaluate inter-observer or intra-observer variability. Further exploration with longer follow up and an external validation cohort will be useful to validate our results.

## Conclusions

In conclusion, radiologic complete response on MRI after NAC in patients with ER-positive/HER2-negative tumors is associated with an excellent outcome. In the case of residual enhancement on MRI after NAC, the largest diameter of late enhancement may be helpful to identify patients who may need additional treatment.

## Abbreviations

AC, doxorubicin and cyclophosphamide; AD; doxorubcin and docetaxel; CR, complete remission; ddAC, (dose-dense) cyclophosphamide and doxorubicin; ER, estrogen receptor; HER2, human epidermal growth factor receptor 2; HR, hazard ratio; IHC, immunohistochemical assessment; MIP, maximum intensity projection; MRI, magnetic resonance imaging; NAC, neoadjuvant chemotherapy; pCR, pathological complete remission; PR, progesterone receptor; RFS, recurrence-free survival; ypT0/is, no residual invasive tumor in the breast; ypT0/isypN0, no residual invasive tumor in breast and axilla; ypT < mic, few scattered tumor cells in the breast
